# Paraneoplastic hypocalcemia-induced heart failure in advanced breast cancer: A case report and literature review

**DOI:** 10.3892/ol.2015.3326

**Published:** 2015-06-05

**Authors:** ALBERTO FAROLFI, CRISTIANO FERRARIO, MICHELE AQUILINA, LORENZO CECCONETTO, ANDREAS TARTAGLIA, TONI IBRAHIM, LUIGI SERRA, DEVIL OBOLDI, MAURIZIO NIZZOLI, ANDREA ROCCA

**Affiliations:** 1 Department of Medical Oncology, Istituto Scientifico Romagnolo per lo Studio e la Cura dei Tumori (IRST) IRCCS, 47014 Meldola (FC), Italy; 2 Cardiology Unit, Istituto Scientifico Romagnolo per lo Studio e la Cura dei Tumori (IRST) IRCCS, 47014 Meldola (FC), Italy; 3 Endocrinology and Metabolism Unit, Morgagni-Pierantoni Hospital, 47121 Forlì (FC), Italy; 4 Pathology Unit, Morgagni-Pierantoni Hospital, 47121 Forlì (FC), Italy; 5 Radiology Unit, IRST IRCCS, 47014 Meldola (FC), Italy

**Keywords:** bone metastasis, breast cancer, heart failure, hypocalcemia, paraneoplastic syndrome

## Abstract

Hypocalcemia is an uncommon clinical symptom of patients with malignant tumors, and a number of factors may be involved in its development. The present study describes the case of a 67-year-old Caucasian female, presenting with severe refractory hypocalcemia and heart failure. The patient was subsequently diagnosed with breast cancer and bone metastases. The paraneoplastic origin of the syndrome was confirmed by its complete resolution once the tumor responded to specific antineoplastic treatments, comprising weekly paclitaxel and aromatase inhibitor administration. The present case report suggested the need for greater awareness of the possibility of paraneoplastic hypocalcemia in breast cancer patients, and suggested that this condition may also contribute to the occurrence of heart failure. The mechanisms potentially responsible for this event were discussed and a brief review of the literature presented.

## Introduction

Hypercalcemia is one of the most common paraneoplastic syndromes, occurring in up to 30% of cancer patients (5–10% with bone metastases) during the course of the disease (1). By contrast, hypocalcemia is a rare event, and is significantly more rare in neoplastic disease. Several factors may be involved in the development of hypocalcemia in cancer patients, including hypoalbuminemia, surgical and infiltrative hypoparathyroidism, hypomagnesemia, vitamin D deficiency, renal failure, massive cell lysis, drug effect, sepsis and osteoblastic metastases (2).

Hypocalcemia derived from bone lesions has mainly been reported in the advanced stages of prostate cancer, resulting from the influx of calcium into bone following abnormal increases in bone formation (3). In such patients, hypocalcemia is typically mild and clinical signs are rare (affecting ~0.9% of patients) (4); however, cases of severe hypocalcemia have occasionally been reported (5–7). In breast cancer patients, hypocalcemia is markedly more unusual than in prostate cancer, and appears to be more frequently associated with the abnormal endocrine activity induced by low parathyroid hormone (PTH) levels (2,8–10).

The present report describes a case of severe hypocalcemia and associated heart failure observed as a clinical presentation of subsequently diagnosed bone-metastatic breast cancer in a patient with normal PTH values.

## Case report

A 67-year-old female was admitted to Morgagni-Pierantoni Hospital (Forlì, Italy) in April 2013, with symptoms of severe fatigue and dyspnea. Signs of heart failure were identified, including lower leg edema and liver stasis, and a chest X-ray revealed bilateral pleural effusion. Blood tests revealed severe normochromic and normocytic anemia [hemaglobin (Hb) 6.1 mg/dl] and moderate thrombocytopenia (53,000 platelets/mm^3^). Elevated N-terminal of the prohormone brain natriuretic peptide (NT-proBNP) levels were detected (4237 pg/ml; normal values, <900 pg/ml) and electrocardiography (ECG) revealed normal left ventricular dimensions and a 59% ejection fraction. Blood tests also indicated severe hypocalcemia [4.2 mg/dl (1.05 mmol/l), corrected calcium 4.7 mg/dl (1.2 mmol/l)], albumin 34.1 g/l (range, 35–50 g/l) and normal PTH values (41 ng/l; range, 15–60 ng/l). Creatinine levels were 0.6 mg/dl, with an estimated creatinine clearance of 95 ml/min, while 25-hydroxyvitamin D was 4.9 µg/l (range, 20–50 µg/l). No clinical signs of latent tetany were detected.

Treatment for heart failure commenced with administration of a β-blocker (bisoprolol, 1.25 mg daily; Bayer AG, Leverkusen, Germany), an angiotensin-converting enzyme inhibitor (ramipril, 2.5 mg daily; Sanofi S.A., Paris, France) and a diuretic (furosemide, 25 mg daily; Sanofi S.A.). Calcium and vitamin D supplements were also administered intravenously and orally.

Clinical examination identified a poorly defined mass of ~6 cm in the right breast, with partial nipple retraction. Mammography highlighted bilateral reticular enhancement of the gland with skin thickening, and a biopsy revealed infiltrating lobular carcinoma, with estrogen receptor (ER) 100%, progesterone receptor (PR) 50%, proliferation index (Mib1) 5% and no amplification of human epidermal growth factor receptor type 2 (HER2).

**Figure 1.**
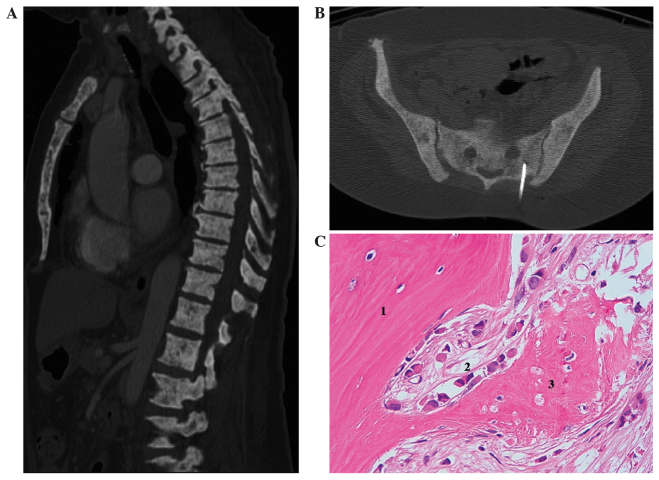
Clinical characteristics of the 67-year-old Caucasian female patient. (A) Computed tomography (CT) scan identified massive calcium deposits in the bone. (B) Subsequent bone biopsy (shown in CT image) revealed bone marrow metastases from lobular carcinoma. (C) 1, Normal trabeculae of spongy lamellar bone; 2, Bone marrow infiltrated by cancer cells; 3, Novel bone apposition with activated osteoblasts (hematoxylin and eosin staining; magnification, x10).

A computed tomography (CT) scan revealed moderate pleural effusion and bilateral axillary adenopathies, mild ascites associated with thickening of peritoneal fat and massive calcium deposits in the bones (Fig. 1A). The unusual bone images were initially interpreted as metabolic syndrome secondary to low or absent PTH production. However, given the patient's overall clinical situation, the possibility of bone metastases was not ruled out. Due to the unusual radiological features of the bone lesions, a bone biopsy was also performed (Fig. 1B), revealing massive bone marrow metastases as a result of lobular carcinoma of the breast (ER, 100%; PR, 0%; Mib1, 1% and HER2, not amplified), with associated bone-thickening (Fig. 1C).

**Figure 2.**
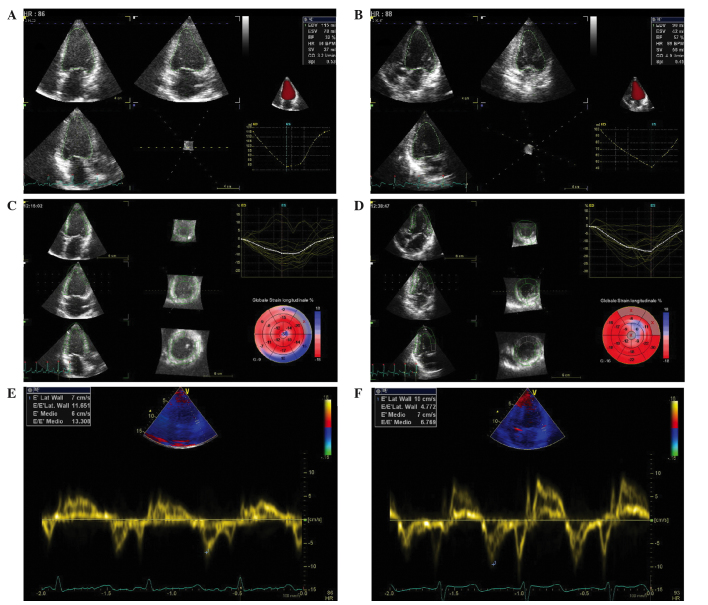
Depression of cardiac function during severe hypocalcemia (3.7 mg/dl) and restoral following serum calcium normalization. (A) 3D echocardiography indicated a 3D-LVEF of 32% (normal, >55%) with left ventricular dilatation. (B) Volume normalization and 3D-LVEF of 57% following treatment. (C) GLS reduction at speckle tracking (−9%). (D) Recovery following calcium normalization (GLS, −16%). (E) Abnormal E/E' ratio (13.3, normal values <8; non-invasive reliable estimation of the degree of ventricular filling pressures) detected by 3D-tissue Doppler. (F) Normalization following calcium restoration (E/E', 6.7). GLS, global longitudinal strain; 3D, 3-dimensional; LVEF, left ventricular ejection fraction.

The patient was subsequently admitted to the Department of Medical Oncology of the Istituto Scientifico Romagnolo per lo Studio e la Cura dei Tumori (IRST) Istituto di Ricovero e Cura a Carattere Scientifico (IRCCS). Despite the administration of calcium (40 mEq/day) intravenously, in addition to the 2.0 g administered orally during the previous month in Morgagni-Pierantoni Hospital, the patient's serum calcium decreased to 3.7 mg/dl (0.93 mmol/l), with a corrected calcium level of 4.1 mg/dl (1.03 mmol/l). An ECG indicated a prolonged QT-interval (489) and three-dimensional (3D)-echocardiography revealed a dilated and hypokinetic left ventricle, with a reduced 3D-left ventricular ejection fraction (3D-LVEF) of 32% (Fig. 2A), pulmonary hypertension (44 mmHg) and reduced global longitudinal speckle tracking strain (GLS, −9%) (Fig. 2B and C). 3D-tissue Doppler imaging also revealed an abnormal degree of ventricular filling pressure, estimated by the transmitral flow velocity to mitral annular velocity ratio (E/E', 13.3; normal values, <8). The NT-proBNP value had also increased to 15388 pg/ml.

The PTH was 51 ng/ml (range, 15–65 ng/ml) and calcitonin <2.00 ng/l, with a 25-hydroxyvitamin D value of 5.2 µg/ml. Serum phosphate was 5.3 mg/dl (range, 2.70–4.50 mg/dl), magnesium 1.96 mg/dl (range, 1.60–2.60 mg/dl), sodium 136 mMol/l (range, 136–145 mMol/l) and potassium 4.6 mMol/l (range, 3.5–5.1 mMol/l). Electrolyte values in a 24-h urine test were unremarkable (calciuria, <1 mg/dl). Following approximately one week in our department, during which the patient was treated with calcium supplements (60 mEq/day administered intravenously and 1.5 g administered orally), serum calcium was 5.8 mg/dl (1.45 mmol/l), corrected calcium 3.0 mg/dl (0.75 mmol/l) and serum phosphate 5.3 mg/dl (range, 2.7–4.5 mg/dl). Treatment with 0.5 mcg calcitriol twice a day was continued.

The patient subsequently began a course of oral aromatase inhibitor (letrozole; 2.5 mg daily) and weekly paclitaxel (taxane; 90 mg/m^2^ on days 1, 8 and 15 of a 28-day cycle) treatment.

**Figure 3.**
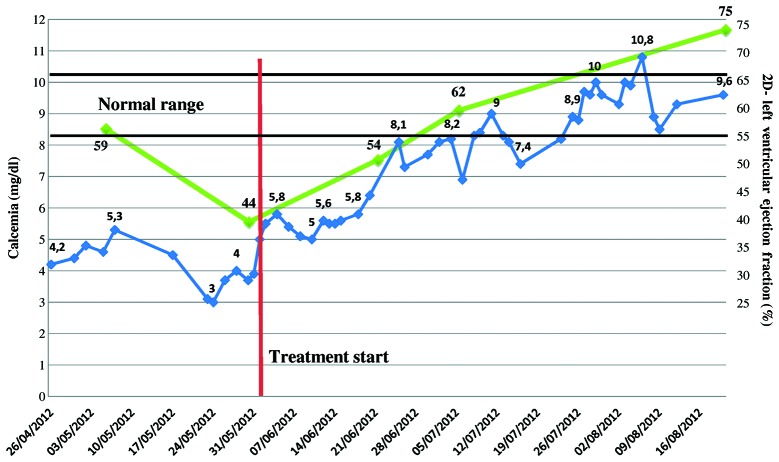
Antineoplastic treatment normalizes calcium and LVEF. Temporal trends in calcium (blue) and LVEF (green). The red line indicates the initiation of antineoplastic treatment, while the black lines delimit the normal serum calcium range, 8.6–10.2 mg/dl. Normal LVEF >55%. LVEF, left ventricular ejection fraction.

Following 3 weeks of treatment with paclitaxel and letrozole, calcium values had almost returned to normal (8.1 mg/dl), NT-proBNP had decreased to 1927 pg/ml, the QT-interval had fallen to 467 and a 3D-echocardiography indicated improved systolic function (3D-LVEF, 57%) with a reduction in pulmonary arterial pressure (30 mmHg). GLS had increased from −9 to −16% and E/E' had normalized (6.7) (Fig. 2D–F). As treatment continued, the calcium levels and LVEF continued to markedly improve (Fig. 3).

The patient subsequently developed respiratory symptoms diagnosed as acute respiratory distress syndrome (ARDS), likely a result of an atypical reaction to paclitaxel. Taxane treatment was thus suspended and treatment proceeded with letrozole. Following resolution of the ARDS, the patient's clinical condition continued to improve, with a concomitant clinical reduction in the size of the right breast mass. Intravenous calcium supplements were gradually discontinued and treatment with zoledronic acid commenced, without complications.

To date, the patient remains asymptomatic and the most recent CT scan indicated an improvement on the previous radiological findings. Treatment with letrozole and oral calcitriol and calcium continues.

Written informed consent for the publication of this case report was obtained from the patient.

## Discussion

Severe hypocalcemia presents mainly with paresthesia, neuromuscular irritability and tetany. Other less frequent symptoms include cardiovascular complications, often characterized by ECG alterations (prolongation of the QT interval, which may induce *torsades de pointes*) and heart failure, particularly if calcium plasma levels are notably low (11).

The most frequent causes of symptomatic hypocalcemia in patients with malignancies are bisphosphonate therapy and tumor lysis syndrome (12); however these conditions were not applicable to the patient in the current study. Ectopic secretion of calcitonin, a paraneoplastic syndrome typically associated with lung cancer (13), was also excluded as normal serum levels of this hormone were present. Oncogenic osteomalacia is another rare paraneoplastic syndrome characterized by hypophosphatemia, low 1,25-dihydroxyvitamin D serum levels and typical radiological features (14). This syndrome is thought to be induced by humoral factors produced mainly by mesenchymal tumors that inhibit renal tubular resorption of phosphate and 1α-hydroxylation of 25-hydroxyvitamin D (15). The patient did not exhibit hypophosphatemia or hyperphosphaturia, thereby excluding this syndrome as a potential cause of the observed symptoms.

To the best of our knowledge, this is the first described case of paraneoplastic hypocalcemia-induced heart failure with normal PTH values (2,16). Frequently reported in advanced prostate cancer, hypocalcemia is suggested to be a consequence of osteoblastic metastases inducing increased calcium uptake in the bones, which may result in secondary hyperparathyroidism (12,17). This is due to calcium-sensing receptors in parathyroid cells that detect very small changes in the plasma calcium-ion concentration, triggering the release of PTH when serum calcium is decreased (18). In the kidney, PTH increases tubular calcium resorption and induces the hydroxylation of 25-hydroxyvitamin D to the active metabolite of vitamin D, 1,25-dihydroxyvitamin D. Within the bone, resorption is enhanced, facilitating greater entry of calcium to the plasma (19). In patients with chronic kidney disease, low serum calcium induced by renal calcium loss results in secondary hyperparathyroidism (20,21). Therefore, chronic hypocalcemia should result in a concomitant increase in PTH secretion.

Hypocalcemia-induced heart failure has previously been associated with idiopathic or postsurgical hypoparathyroidism, vitamin D deficiency and celiac disease (22–24). In the cardiac muscle, calcium functions as a direct central mediator of electrical activation and ion channel gating, has a crucial role in the mediation of excitation-contraction coupling and is also necessary for epinephrine-induced glycogenolysis (25).

Notably, there was a complete restoral of heart function and pulmonary artery pressure in the patient following normalization of serum calcium concentration. However, the precise mechanisms underlying the development of heart failure in the event of extracellular calcium depletion remains to be elucidated (26). In the present study, although cardiac depression was identified, the patient demonstrated no weakness or abnormal muscle tone.

It was previously demonstrated that hypocalcemia is the only independent predictive factor for left ventricular diastolic dysfunction in patients with chronic kidney disease (27). Accumulating evidence has suggested that PTH stimulates aldosterone secretion by enhancing calcium concentration within the cells. Elevated aldosterone levels in primary and secondary hyperaldosteronism are accompanied by enhanced urinary and fecal loss of magnesium and calcium. The resulting decrease in serum calcium concentration further stimulates production of PTH, which induces amplification of adrenal aldosterone synthesis. Excess PTH subsequently induces calcium overload and oxidative stress in cardiomyocytes and exacerbates the reduction in intra-mitochondrial adenosine triphosphate levels, resulting in necrotic cell death and myocardial fibrosis (28,29).

By contrast, the patient in the present study exhibited normal PTH values, which perturbed diagnosis of the underlying cause of the severe hypocalcemia. The normalization of serum calcium levels following antineoplastic treatment, associated with a clinical response, confirmed the paraneoplastic nature of the condition, which was likely induced by hungry osteoblastic bone metastases. However, it may also be hypothesized that the tumor cells released PTH-related peptide (PTH-rP), which functions as a decoy hormone for parathyroid glands; not activating osteoclasts and bone resorption, but inhibiting cardiac function (30). PTH receptors have also been identified in cardiomyocytes (28). Whether the mechanism was via normalization of serum calcium levels or reduction in PTH-rP release, the clinical response resulted in improved heart function.

In conclusion, the results of the present study indicated that the patient likely experienced an effect known as ʻhungry-bone syndromeʼ, which induced extreme, prolonged hypocalcemia and eventually resulted in heart failure (31). The response to antineoplastic treatment resulted in decreased tumor load, confirmed by lower tumor marker values and a reduction in the size of the breast mass, which consequently resulted in decreased calcium utilization by the metastatic process and an improvement in heart function.
